# A Catastrophic Outcome of Ineffectual Episiotomy Causing Acquired Vaginal Atresia: A Case Report

**DOI:** 10.7759/cureus.39574

**Published:** 2023-05-27

**Authors:** Indira Prasad, Shivangni Sinha, Smita Singh, Mukta Agarwal, Afra Asiya

**Affiliations:** 1 Obstetrics and Gynaecology, All India Institute of Medical Sciences, Patna, Patna, IND

**Keywords:** routine episiotomy, case reports, adhesiolysis, vaginismus, vaginal atresia

## Abstract

Episiotomy is an aid done to prevent perineal tears, which may involve the anal sphincter and rectum. However, if not given judiciously, this can result in an increase in morbidity in patients. We present a case report of two young females who presented to our outpatient department with a complaint of vaginismus after their previous vaginal deliveries. The first patient had partial vaginal atresia and the second patient had complete vaginal atresia after an episiotomy repair. The complication arose due to mismanaged episiotomy repair that had a severe impact on their physical, sexual, and psychological well-being. They both underwent vaginal stricture release and adhesiolysis showed satisfactory outcomes during their follow-up.

Though not recommended, prophylactic episiotomy continues to be widely performed. The approach adopted during the operative delivery stays unclear, as episiotomy execution is likely to be impacted by the physician's working environment, as well as maternal and fetal circumstances. Trained execution at rural or urban and private or public facilities is the need of the hour. Counseling regarding prophylactic or emergency episiotomy and its consequences during labor should be considered as a part of their antenatal care.

## Introduction

Gynatresia is a constriction of the female genital tract, which often leads to occlusion or obstruction produced by a thin membrane in any region of the tract. It can be inherited or acquired. Acquired gynatresia is a rare complication, with an incidence of around 7/1,000 women, but it is relatively common in Africa, especially Nigeria, with the most common causes being the insertion of caustic vaginal pessaries, birth injury, and complications of vesicovaginal fistula repair [1, 2]. The risk of perineal complications can increase up to 30% if episiotomy is resorted to routinely [3]. We present a case report of two patients in their 20s who had episiotomy-assisted vaginal delivery that had deleterious effects on their sexual functioning and behavior.

## Case presentation

Case 1

A 24-year-old patient presented to the outpatient department of a tertiary care center with complaints of inability to conceive. She was parity 1 with no live issues. In early 2019, she had her first conception. It was a spontaneous conception and had no associated comorbidities. Her antenatal period was uneventful. At term gestation, she went into spontaneous labor. During the course of delivery, an episiotomy incision was given by their local dai (traditional birth attendants comprising primary health care providers during pregnancy and childbirth in rural areas of India). Following a failed labor trial at home, the patient was referred for institutional delivery in view of obstructed labor. Subsequently, a caesarian section was performed in a local private nursing home, and the episiotomy was repaired. The delivered baby, however, did not cry at birth. The patient and family returned back to their homeplace. During her postpartum phase, the patient complained of vaginal discomfort and difficulty in cohabitating together. She failed to seek medical care and presented to our hospital with a wish to conceive. On detailed history, the patient complained of vaginismus and feeling smaller vaginal introitus than before by her spouse.

**Figure 1:**
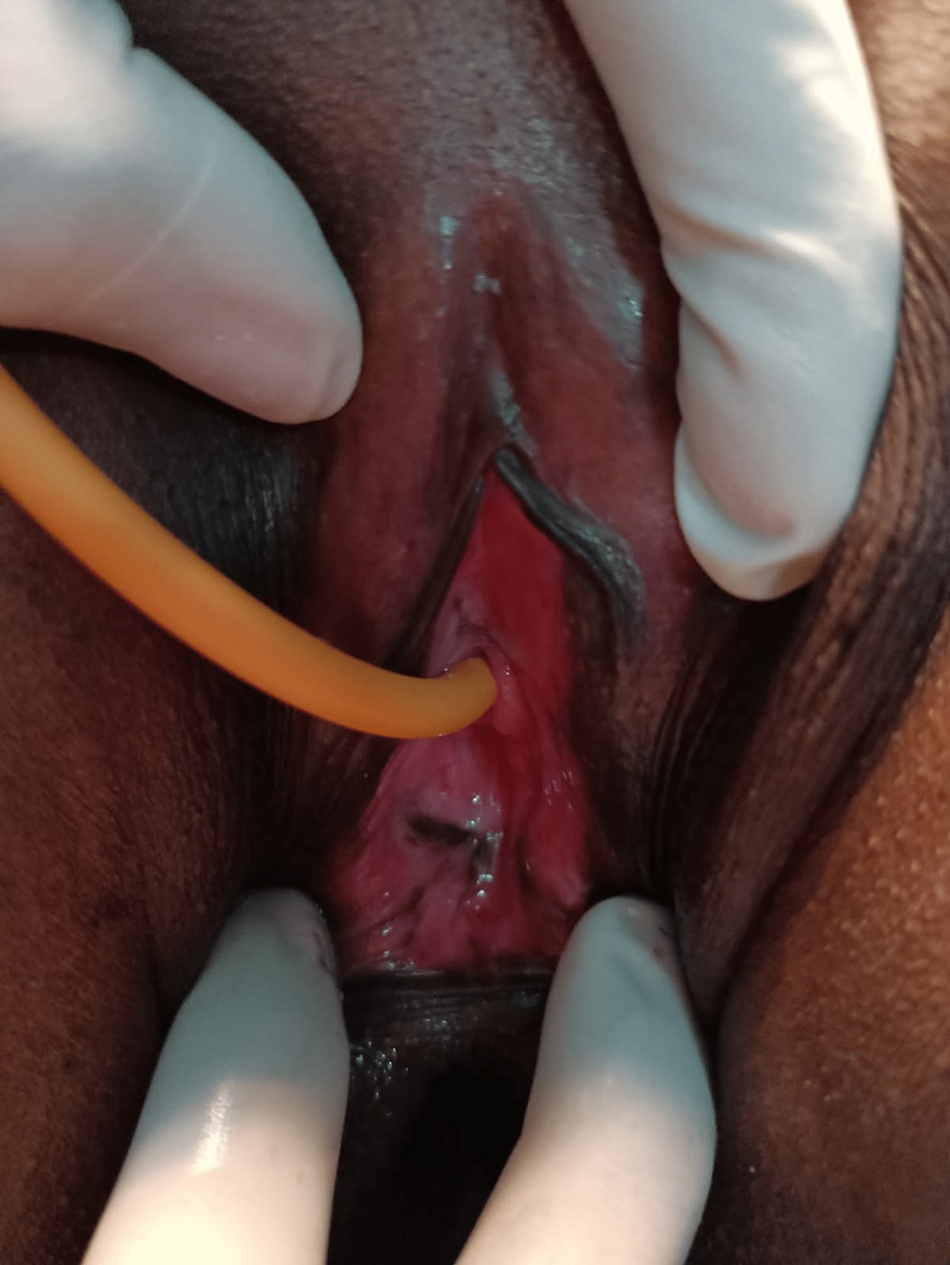
Pre-operative vaginal orifice with a rim of fibrosed tissue

**Figure 2:**
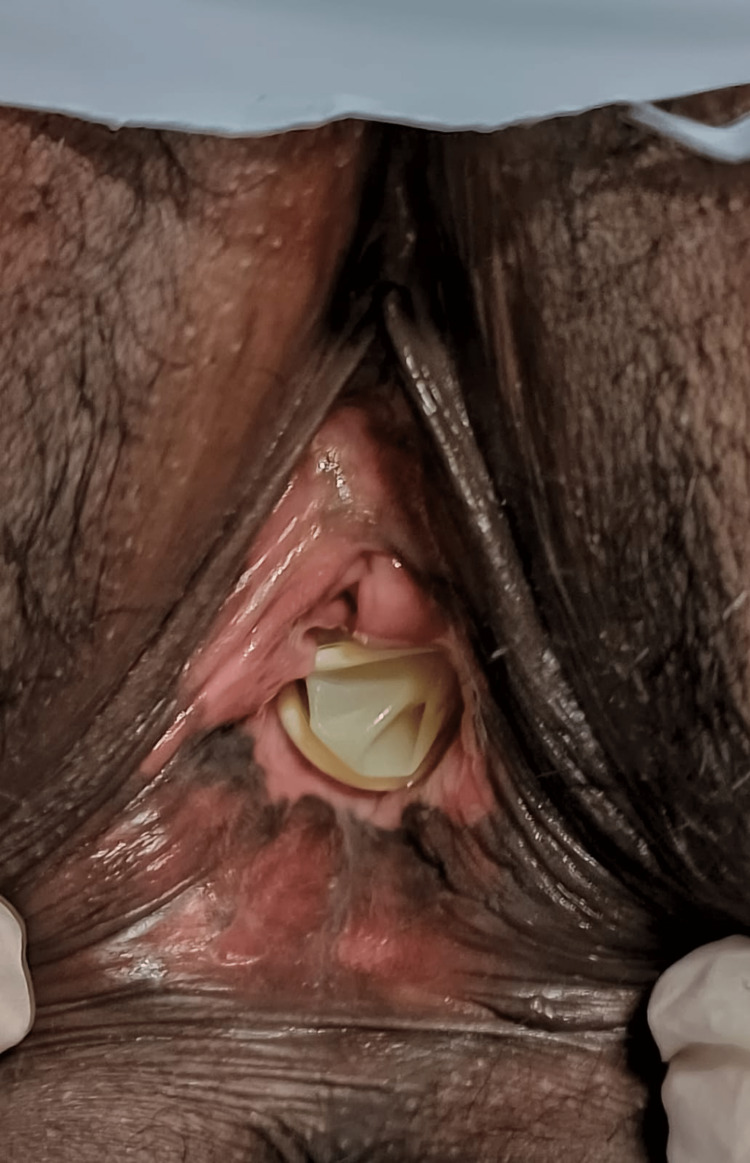
Post-operative image with mold in-situ

The patient’s general examination was within normal limits, and on local examination, the external genitalia appeared normal. Per speculum examination, a pediatric Sims bivalve speculum could be negotiated with difficulty, showing the vaginal orifice to be narrow; the cervix appeared pulled up and healthy. On per vaginal examination, the vaginal orifice was found to be around 1.5 cm wide, with a rim of fibrosed tissue felt all around. The vaginal length was approximately 3-4 cm, with the cervix felt at the tip of a finger. After performing a thorough evaluation, obtaining written informed consent, and conducting counseling, the patient was diagnosed with acquired vaginal atresia and was planned for vaginal stricture release under anesthesia. Through sharp dissection, the fibrotic ring was released pre-operatively. A dental cake mold covered with Surgicel was placed in situ. Mold change was done on Day 7, followed by discharge from the hospital. The patient’s postoperative hospital stay was uneventful. She was advised to use mold regularly and asked to follow up after six weeks. At the time of being discharged from the hospital, she was advised abstinence for two months. During her follow-up, the symptoms had relieved. Figures 1-2 present preoperative and postoperative images respectively.

 Case 2

A 28-year-old patient presented to our outpatient department of obstetrics and gynecology in a teaching institute with complaints of experiencing dyspareunia and vaginismus for six months. She had a history of delivering a full-term, 3.8 kg female infant at a local public healthcare center (PHC) 10 months ago. She had gone into spontaneous labor, and as she also had a history of prolonged labor and difficulty in delivering, an episiotomy was performed. The patient complained of vaginal discomfort for which a sitz bath and the local application of medications were advised. The patient was discharged immediately after delivery. She failed to follow up as advised during her postpartum period. She had been breastfeeding her child and had been experiencing lactation amenorrhea since delivery.

**Figure 3:**
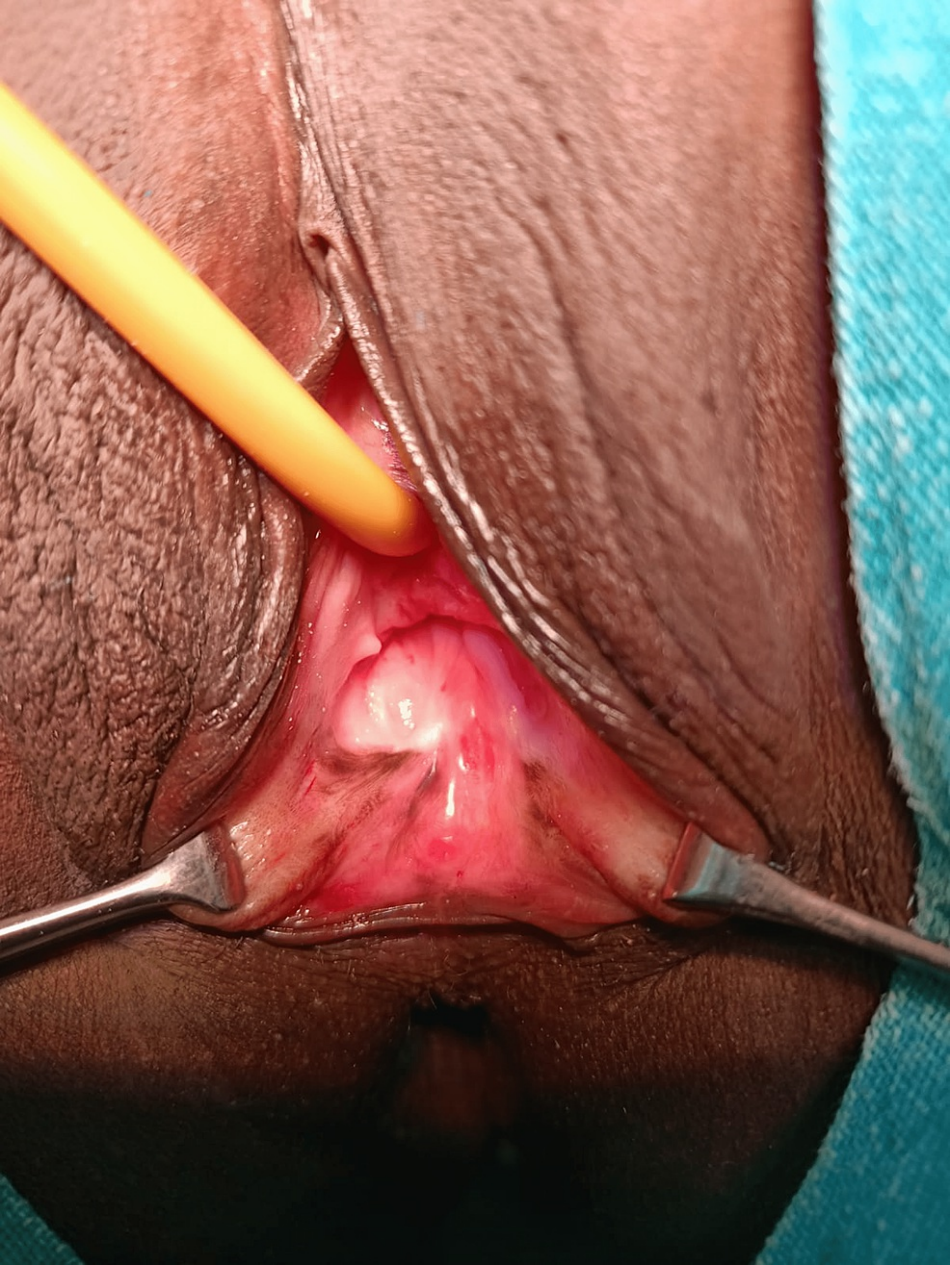
Pre-operative picture with vaginal dimple

**Figure 4:**
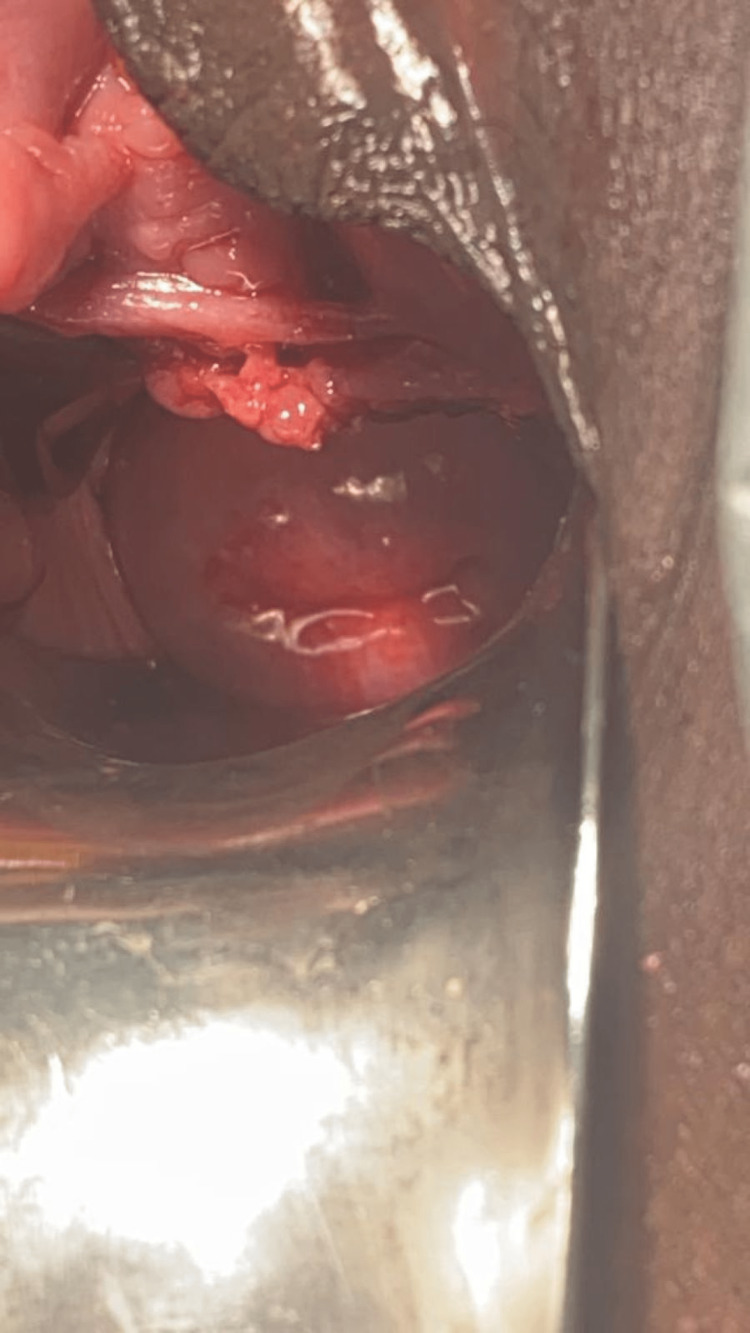
Post-operative image following hematocolpos drainage with normally seen cervix within

On physical examination, the patient was noted to be of average build with stable vitals. The local examination suggested vaginal stricture, a 0.5 cm vaginal dimple with no patent opening. After performing a thorough examination, conducting counseling, and obtaining written informed consent, the patient was scheduled for vaginal stricture release. Pre-operatively, the strictures were released through both sharp and finger dissection and successive dilatation of the vaginal canal. Hematocolpos of around 60 ml was drained and sent for culture sensitivity. A dental cake mold with surgical was inserted per-vaginally post-operatively. The patient’s postoperative hospital stay was uneventful. Her vaginal mold change was done on Day 7 postoperatively, and she was discharged in stable condition. The patient was advised to follow up six weeks after the operation. Figures 3-4 show the preoperative and postoperative images.

## Discussion

Episiotomy is a surgically planned incision on the perineum and the posterior vaginal wall during the second stage of labor to enlarge the vaginal introitus to facilitate easy, safe delivery and prevent overstretching of perineal muscles and fascia. Perineal trauma is a prevalent complication of standard vaginal birth, affecting 53-75% of patients worldwide [4]. Episiotomy is still done in around 12% of US births, although it is not recommended as a standard practice in 2006 [5]. According to a study in 20 European countries, the rates of episiotomy practice ranged from 3.7% in Denmark to 75.0% in Cyprus, but this figure is still higher in developing countries like Africa, Asia, and Latin America compared to the aforementioned countries where episiotomy is practiced routinely or liberally [6].

For years, episiotomy was thought to be a controlled modality to provide larger vaginal space during childbirth. Experts believed that a prophylactic incision would heal better than a natural tear. Today, however, research suggests that episiotomy that is routinely carried out does not prevent these problems; it may even cause worse tearing. Moreover, it doesn't prevent complications like urinary or bowel incontinence or painful sex that may be caused by a natural perineal tear. In 2006, the American College of Obstetricians and Gynecologists (ACOG) recommended against resorting to routine episiotomy [7].

The NICE clinical guideline on intrapartum care for healthy women and babies recommends performing an episiotomy only if there is a clinical need, such as in the case of an instrumental birth or suspected fetal compromise. An episiotomy should be performed mediolaterally, using Episcissors-60, starting at the vaginal fourchette and directed toward the right side at the time of crowning. The angle of the cut to the vertical axis is recommended to be 45-60 degrees [7].

Recovery after episiotomy could be uncomfortable. Occasionally, the incision may be larger than a natural tear. A meta-analysis of 16 studies (3,133 women) concluded that episiotomy was associated with the highest rate of perineal pain and incidence of dyspareunia on the resumption of sexual activity [8]. One study showed that patients who complained of dyspareunia during sexual intercourse at three months post-partum typically had an episiotomy during delivery [3]. Perineal care in the form of warm or cold shallow baths (sitz baths), locally applying medicated creams or sprays like Dermoplast, and medication, such as acetaminophen with codeine, form an integral part of puerperal care. The incision wound should be kept clean and dry.

Both the techniques and materials used for perineal repair widely vary between person individual and maternity units. The rationale for choosing the suturing technique appears to be based on how the surgeon performing the procedure was first taught. Many countries are putting midwives in the spotlight in order to improve treatment quality, prevent "over-medicalization" during childbirth, and promote resource efficiency. In places where midwifery-led units are operational, midwives are the first point of contact for pregnant women. They help to develop a surveillance mechanism by taking care of most standard childbirths and referring only complicated cases to healthcare facility leads at "midwifery-led care units," which are established at "LaQshya"-certified high case load healthcare centers. Applicants trained under the midwifery program are employed at these units for round-the-clock delivery services; medical colleges have 16-18 midwives, district hospitals have six to eight midwives, and community hospitals have six to eight midwives. The instructional modules are based on the "Essential Competencies for Midwifery Practice (2018 update)," which have been authorized by ICM and WHO guidelines to provide services to pregnant women such as contraception, antenatal care, delivery, postnatal care, and safe abortion. Kapoor and Sultan discovered that a large proportion of the participating clinicians had a poor understanding of perineal anatomy, trauma classification, and satisfaction with perineal management training in a survey of 75 midwives and 75 trainee doctors. Only 20% of trainees and 48% of midwives said their instruction was of "good standard." Many answers to questions about anatomy and perineal surgery were erroneous; for example, most classified a partial or complete tear of the external anal sphincter as “second degree” [9-10].

The Perineal Assessment and Repair Longitudinal Study (PEARLS) is a clinical quality improvement program aimed at improving the assessment and management of persistent trauma in the United Kingdom and around the world [11]. It could be theorized that even if the best suture techniques and materials are used to repair perineal tears, the short‐ and long‐term outcome of the procedure depends on the skill of the practitioner.

Consent to episiotomy is subject to the same level of legal and professional requirements as other interventions of this kind, yet it is often neglected. A positive childbirth experience, according to WHO, is one that meets or exceeds a woman's prior personal and sociocultural values and requirements, such as giving birth to a healthy baby in a clinically and psychologically safe environment with constant practical and emotional support from a birth companion(s) and compassionate, qualified clinical staff. This concept is founded on the assumption that most women prefer physiological labor and birth, as well as a sense of personal accomplishment and control through choices, even when medical interventions are required or desired. In a qualitative study carried out in an urban teaching hospital in London on 15 patients, it was concluded that informed and voluntary consent for episiotomy is not consistently sought, and often takes the form of mere compliance on their part [12]. A genuine choice must be offered to women, after duly informing them about episiotomy’s associated risks, likely consequences, and alternatives.

This case report strongly supports the importance of post-partum care and the need for counseling on episiotomy during antenatal care itself. It should be considered a part of labor care and rightful information on its complications needs to be focussed. Further, couples training and awareness of danger signs should be a part of quality health care.

## Conclusions

Given the need for competent workers to provide superior medical services to 30 million pregnant women each year in India and given the aforementioned challenges on this front, it has become necessary to develop a novel and alternative model for conducting vaginal delivery by professionally trained personnel. The new model should posit a much-improved approach to reproductive, maternal, and neonatal health services in India such that related iatrogenic morbidities can be prevented.
